# Lateral flow immunoassay (LFIA) for the detection of lethal amatoxins from mushrooms

**DOI:** 10.1371/journal.pone.0231781

**Published:** 2020-04-17

**Authors:** Candace S. Bever, Catharine A. Adams, Robert M. Hnasko, Luisa W. Cheng, Larry H. Stanker

**Affiliations:** 1 Foodborne Toxin Detection and Prevention Research Unit, Western Regional Research Center, United States Department of Agriculture, Agricultural Research Service, Albany, California, United States of America; 2 Plant and Microbial Biology Department, University of California, Berkeley, California, United States of America; 3 Produce Safety and Microbiology Research Unit, Western Regional Research Center, Agricultural Research Service, United States Department of Agriculture, Albany, California, United States of America; Universidade Federal de Minas Gerais, BRAZIL

## Abstract

The mushroom poison that causes the most deaths is the class of toxins known as amatoxins. Current methods to sensitively and selectively detect these toxins are limited by the need for expensive equipment, or they lack accuracy due to cross-reactivity with other chemicals found in mushrooms. In this work, we report the development of a competition-based lateral flow immunoassay (LFIA) for the rapid, portable, selective, and sensitive detection of amatoxins. Our assay clearly indicates the presence of 10 ng/mL of α-AMA or γ-AMA and the method including extraction and detection can be completed in approximately 10 minutes. The test can be easily read by eye and has a presumed shelf-life of at least 1 year. From testing 110 wild mushrooms, the LFIA identified 6 out of 6 species that were known to contain amatoxins. Other poisonous mushrooms known not to contain amatoxins tested negative by LFIA. This LFIA can be used to quickly identify amatoxin-containing mushrooms.

## Introduction

**Fig 1:**
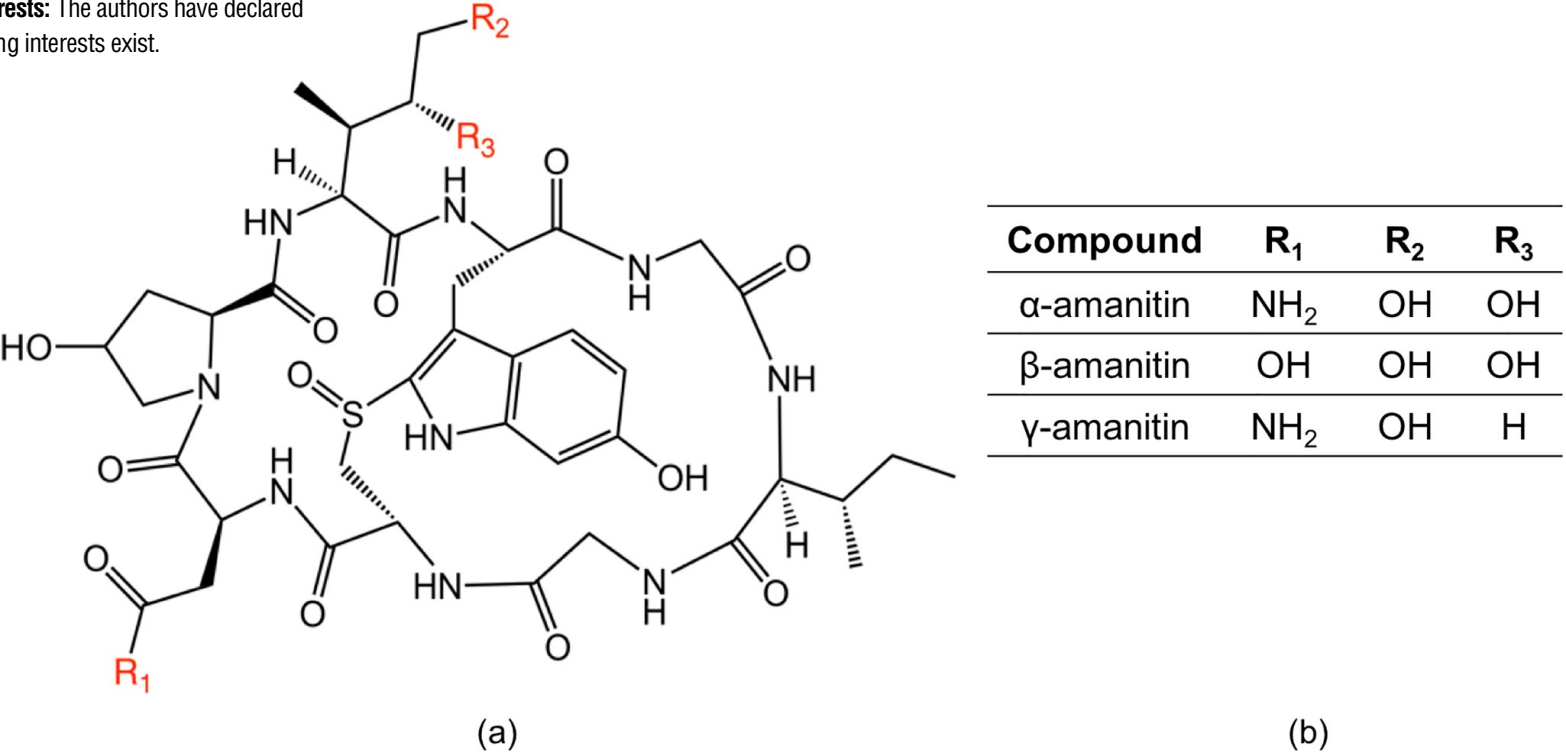
Chemical structures of the amatoxin variants examined in this paper. (a) molecular structure of amanitin. (b) R-group designations for each variant.

Globally, thousands of mushroom poisonings are reported each year [1–9]. Approximately 80% of the mushroom poisonings involve unknown mushroom species. The poisonous mushrooms are often classified based on the toxins involved and the clinical signs they elicit [10]. Most of the lethal cases are attributed to mushrooms that contain amatoxins. Amatoxins are a family of bicyclic octapeptides that are not inactivated by extreme temperatures, pH, cooking, or digestive enzymes in humans. The principal toxins responsible for toxicity are the amanitins (here, amatoxins; Fig 1), most prominently α-amanitin (α-AMA), β-AMA and γ-AMA. They are potent inhibitors of RNA polymerase II, essentially halting protein synthesis in eukaryotes. The human LD_50_ for active amatoxins (estimated as the total content of the major toxic amanitins) in a fresh mushroom is considered to be ~ 0.1 mg/kg [11]. When α-AMA, β-AMA, and γ-AMA were tested individually in mice (via ip injection), the LD_50_s ranged from 0.2–0.8 mg/kg [12, 13]. Amatoxin-containing mushrooms include a few species from the genera *Amanita*, *Galerina*, and *Lepiota* [11].

In addition, there is another class of structurally related cyclopeptide toxins, the phallotoxins. These are produced mainly by *Amanita* species, and debatably by a single *Conocybe* species [11, 14]. Phalloidin, the most well-studied phallotoxin, tightly binds filamentous actin, which prevents depolymerization and ultimately leads to cell death in eukaryotes. Though toxic to eukaryotic cells, phallotoxins are not absorbed through the gastrointestinal tract and thus do not seem to play a role in human mushroom intoxication [13]. Both the amatoxins and phallotoxins are encoded by the cycloamanide gene family and are biosynthetically produced on the ribosome [15]. Ongoing research continues to explore this pathway to understand more about toxin production and regulation.

For expert mycologists, current techniques to identify toxic mushroom species are based on extensive morphological evaluations of the mushroom and knowledge of its habitat. Mushrooms of the same species can vary in appearance at different growth stages and can appear different due to environmental and genetic factors. Many poisonous mushrooms resemble edible wild mushrooms and all genera that contain poisonous mushrooms also include many non-poisonous and edible mushrooms [16]. For instance, *A*. *velosa* is a highly desirable edible wild mushroom, but it can produce pure white forms, which to amateur mycologists may appear similar to the pure white *A*. *phalloides* var. *alba* [17]. The poisonous white mushroom, *A*. *ocreata*, also emerges in California in the same spring season as the edible *A*. *velosa*. Both associate with oak trees and could be confused by the untrained eye. Mature toxic *Amanita* species can also be misidentified as edible *Volvariella volvacea* (paddy straw mushroom) [18] or for edible *Amanita* speciess (i.e., *A*. *hemibapha* and *A*. *princeps*) naturally found in Southeast Asia [19].

Due to the lethality of amatoxins, there is a great need for a field-portable, simple and accurate chemical test to determine the presence of amatoxins in mushrooms or diagnostic samples. Early attempts for a rapid, chemical assay of amatoxins in mushrooms used the Meixner-Wieland test [20]. The Meixner-Wieland test is a simple procedure wherein juice from a fresh mushroom is rubbed onto lignin-containing paper. In the presence of a concentrated acid, a blueish-green color is observed. However, the test also reacts with hydroxylated indoles and therefore is not specific for amatoxins [21]. False positives were reported 19% (63 out of 335) of the time [22]. For mushroom analysis, instrumental methods (e.g., liquid chromatography-mass spectrometry (LC-MS)) are highly sensitive and selective, but require extensive sample pre-treatment and expensive equipment [23–26]. Immunoassays (e.g., enzyme-linked immunosorbent assays, ELISAs) are sensitive and selective, but still require specialized reagents and equipment, and take a few hours to perform [27–32]. However, these same immunoreagents used in an ELISA can be transferable to a lateral flow immunoassay (LFIA) format, which often significantly reduces the assay time and the need for specialized equipment. Previous attempts to generate a LFIA for amatoxin detection utilized a recombinant single chain variable fragment antibody and was used to evaluate spiked mushroom samples [33].

Recently, we generated new high-affinity monoclonal antibodies (mAbs) for the detection of amatoxins. We also demonstrated that rapid (<1 min) extraction of amatoxins from mushrooms was feasible with simple aqueous-based solutions [32]. In this study, we incorporated the new mAbs into a competitive LFIA. After optimization, the LFIA was characterized to determine analyte sensitivity and selectivity, and product shelf-life. We then used this assay to detect toxins from mushroom extracts and, for the mushrooms tested, compared those results to previous descriptions in the scientific literature (i.e., contains amatoxins or not), while a few selected specimens were screened by LC-MS.

## Materials and methods

### Reagents and components

Monoclonal antibody (AMA9G3; American Type Culture Collection Accession number PTA-125922) and hapten-protein conjugates (PERI-AMA-BSA and LB-AMA-BSA) were produced as described earlier [32, 34]. Colloidal gold (40 nm), goat-anti-mouse IgG, PVC backing cards, nitrocellulose membranes, Ahlstrom 243 wick pad, Ahlstrom 8964 sample pad, and Ahlstrom 8980 glass conjugate release pad (Helsinki, Finland) were provided by DCN Diagnostics Inc (Carlsbad, CA, USA). The nitrocellulose membranes consisted of MDI 150 and MDI 90 (Advanced Microdevices, Pvt. Ltd, India), FF120 and FF80 (GE Healthcare, Pittsburgh, PA, USA), and CN95 and CN140 (Sartorius Stedim Biotech, Concord, CA, USA). Solutions were dispensed using an XYZ3060 Dispensing Platform (BioDot, Irvine, CA, USA) equipped with a Frontline contact dispenser for the antigen and an AirJet dispenser for the antibody-gold conjugates.

The inhibitors tested were α-AMA (≥95%, Funite, Ann Arbor, MI, USA), β-AMA (≥98%, Funite), γ-AMA (≥90%, Enzo Life Sciences, Farmingdale, NY, USA), microcystin-LR (≥95%, Enzo), nodularin (≥95%, Enzo), phalloidin (>90%, Enzo), phallacidin (≥85%, Sigma, St. Louis, MO, USA), pysilocybin (>99%, Cerilliant, Round Rock, TX, USA), muscimol (>99%, Abcam, Cambridge, MA, USA), and ibotenic acid (>98%, Abcam). The remaining reagents were purchased from Fisher (Waltham, MA, USA) or Sigma, unless specified.

All wild mushroom samples were collected from the Point Reyes National Seashore (#PORE-2017-SCI-0054), obtained from local fungal fairs, or provided by generous mycologists. Most (all but 6) of the mushrooms sampled in this study have been deposited in the UC Berkeley Herbarium for future research access.

### Preparation of the conjugate pad

Anti-amatoxin mAb AMA9G3 was conjugated to 40 nm colloidal gold. A checkerboard titration of both pH (6, 7, 8 and 9) and antibody concentration (0, 1, 2, 4, 6, 8, 10 and 12 μg/mL) was used to determine the optimal amount of antibody required to stabilize colloidal gold particles. First, a solution of colloidal gold (OD_540_ = 1) was prepared in borate buffer (10 mM) at each pH (6, 7, 8, and 9) and added to the wells (0.2 mL/well) of a low protein binding microtiter plate. Next, for each pH level, aliquots of antibody were added to achieve the desired final concentrations and then incubated for 5 min at room temperature. A solution of 10% NaCl (20 μL/well) was added and the change in color was assessed. The wells exhibiting no color change provided a stable conjugate. The conditions that permitted the lowest antibody concentration to stabilize the gold were used to produce a larger batch of antibody-gold conjugates. After conjugation, the particles were blocked with 10% bovine serum albumin (BSA) for 30 mins at room temperature, and then centrifuged at 15,000 x g for 20 mins at 4 °C. The pellet was resuspended in borate buffer (50 mM borate, 1% BSA, pH 9) and adjusted to a final OD_540_ of 10. When needed for half strip testing, 5 μL of particles were added to 45 μL of phosphate buffered saline (PBS; 10 mM phosphate, 138 mM NaCl, 2.7 mM KCl, pH 7.4) containing 1% BSA and 0.25% Tween-20, adjusted to pH 8. When used for spraying onto the conjugate release pad, sucrose (10% final) and trehalose (2% final) were added.

For preparation of the conjugate release pad, conjugate pads were first blocked (50 mM Borate, 1% BSA, and 0.25% Tween-20, pH 8) by complete immersion into solution to allow saturation and then dried for 2 hours at 40 °C. Antibody-gold conjugate was sprayed onto the pad at 10 μL/cm and dried for 1 hour at 40 °C.

### Immobilization of the capture reagents onto nitrocellulose membranes

Half strips, consisting of a nitrocellulose membrane and a wick adhered to a backing card, were constructed to determine the ideal antigen and nitrocellulose combination. Two different antigens (PERI-AMA-BSA and LB-AMA-BSA conjugates) were dispensed as test lines onto six different nitrocellulose membranes. PERI-AMA-BSA was coated at 11 mg/mL and LB-AMA-BSA was coated at 1 mg/mL in PBS. Control lines were coated with goat-anti-mouse polyclonal antibodies at 1 mg/mL in PBS. The different nitrocellulose membranes were: MDI 150, GE FF120, GE FF80, MDI 90, Sartorius CN95, and Sartorius CN140. The membranes were dried for 1 hour at 40 °C and when assembled, the wicking pad (21 mm) overlapped the nitrocellulose membranes (25mm) by ~2mm. To visualize, equal aliquots of antibody-gold nanoparticles were placed into the bottom of test tubes and each membrane type was dropped into the solution and run for approximately 10 minutes.

### Preparation and assembly of the lateral flow strips

Full strips were assembled using CN95 coated with antigen LB-AMA-BSA at 0.5 mg/mL. The antigen was applied at 10 μL/cm and then dried for 1 hour at 40 °C. Full strips (4 mm in width) consisted of a 60 mm backing card, a 15 mm sample pad, 10 mm conjugate pad, 25 mm nitrocellulose membrane, and a 21 mm wicking absorbent pad. Fully assembled strips were stored at room temperature in sealed pouches with desiccant, until needed. Full strips were tested both inside and outside of a cassette and no aberrant reactions were observed with each format. For all remaining experiments, full test strips were tested in round-bottom glass test tubes or in wells of a 96 well microtiter plate without the use of a cassette.

### Analytical detection of α-AMA, β-AMA, and γ-AMA by lateral flow immunoassay (LFIA)

The analytical cut-off value was defined as the amount of toxin that just causes complete disappearance of the test line. To determine the cut-off value for α-AMA, β-AMA, and γ-AMA, a set of eight solutions ranging from 0.1 to 10 ng/mL were prepared in PBS. For β-AMA, additional concentrations were tested ranging from 1 to 2000 ng/mL. For each test concentration and the blank containing only buffer, 100 μL of the solution was added to the test strip at the conjugate pad. Each sample was tested in triplicate. The intensity of the lines was resolved by 10 minutes. If no control line appeared, the test was determined to be invalid. The strips were visualized by two independent readers recording a visual score of the test line intensity (0–6; 0 = no color, 1 = barely visible (faint), 2 = weak color, 3 = moderate color, 4 = moderately strong color, 5 = strong color, 6 = very strong color) and by taking a digital photograph of the test strips. Photographs were acquired by a Nikon SLR camera equipped with an LED ring light (B&H Foto and Electronics Corps, New York, NY, USA) for even lighting. The digital image was analyzed with ImageJ software (NIH, Bethesda, MD, USA). Images were contrast enhanced (default setting of 0.3%) and boxes of consistent size were used to integrate the test line’s pixel value. Pixel values were inverted by subtracting the measured value from the maximum possible (i.e., 255). The strips were tested in triplicate and the values were expressed as mean ± standard error. The data was plotted using a 4-parameter logistic equation (GraphPad Prism 7; La Jolla, CA, USA).

### Analytical selectivity of the LFIA

The LFIA test strips were tested with a panel of near neighbor chemicals, such as phallotoxins, other cyclic peptides, and other chemicals known to exist in mushrooms, to determine the selectivity of the assay. The chemicals tested were phalloidin, phallacidin, microcystin-LR, nodularin, pysilocybin, muscimol, and ibotenic acid. Each purified chemical was dissolved in deionized H_2_O, then diluted into PBS at relatively high concentrations. Aliquots of these samples were assessed in triplicate. If cross-reactivity (i.e., a disappearance of the test line intensity) was observed, samples were diluted and re-tested at lower concentrations. A visual qualitative reading of either YES (+, positive test) or NO (–, negative test) was performed by two individuals and a digital image of the strip was acquired as described previously.

Cross-reactivity (%) was calculated as follows: ([cut-off value of α-AMA] / [cut-off value of the test inhibitor] x 100.

### Shelf-life testing of the LFIA

The performance of the test strips over time was assessed via accelerated stability studies to simulate enhanced degradation of the product. The assembled strips packaged in foil pouches with desiccant bags were incubated at 45 and 55 °C with ambient humidity. These conditions were selected as they fall within the typical temperature range for testing in vitro diagnostic products [35]. Testing was performed at 0, 4, 7, 15, 22, 26, 37, 44, and 87 days for the strips kept at 45 °C and at 0, 1, 4, 8, 14, 17, 21, 25, 37 and 52 days for the strips kept at 55 °C. On each of the indicated days, a 100 μL aliquot of PBS, 1 ng/mL of α-AMA in PBS, and 10 ng/mL of α-AMA in PBS, was tested in triplicate for each concentration. Visual score readings were performed by one of three independent readers randomly varied by day. Digital analyses were performed as described previously. Mean values of triplicate measurements from the same dose concentration were compared to the first day values by using a one-way analysis of variance (GraphPad Prism) and a post hoc test (Holm-Sidak method). P-values of less than 0.05 were considered statistically significant. The conversion of accelerated time to standard day was calculated using the Arrhenius equation using a Q_10_ factor of 2.6 [35].

### Mushroom analysis

Whole mushroom specimens were identified by expert mycologists and then dried at 45 °C for 24 hours. The specimens included those that were known to contain amatoxins (*A*. *bisporigera*, *A*. *ocreata*, *A*. *phalloides*, *A*. *marmorata*, *Galerina marginat*a, and *Lepiota subincarnata*) and several that were known to not contain amatoxins, but were either closely related (*A*. *augusta*, *A*. *calyptratoides*, *A*. *constricta*, *A*. *gemmata*, *A*. *magniveracuta*, *A*. *novinupta*, *A*. *pantherina*, *A*. *protecta*, and *A*. *velosa*), locally foraged (*Boletus edulis*, *Cantharellus californicus*, *Galerina sideroides*, *Pholiotina gracilenta*, *Pholiotina utricystidiata*, and *Volvariella volvacea*), or contained other gastrointestinal irritants or hallucinogenic toxins *(A*. *muscaria*, *Agaricus californicus*, and *Ag*. *xanthodermus*).

Small portions of the cap of dried specimens were weighed (~10–200 mg) and then placed into a 15 mL Falcon tube containing 1 mL of PBS. The solutions were briefly (<1 min) swirled by hand and then a 100 μL aliquot of the extract was immediately applied to the sample pad of the LFIA test strip, in triplicate. Each sample produced a visual qualitative reading of either YES (+) or NO (–) which was performed by two individuals, and a digital image of the strip was acquired. The absence of a test line indicated the presence of amatoxins or amatoxin-like compounds, while a visible test line indicated no amatoxins were present in the extract. A visible control line indicated the gold-labeled antibody flowed along the test strip and performed appropriately.

To increase the number of mushroom species tested with this LFIA method, an herbarium collection (dried samples, collected up to 20 years ago) was utilized to sample a large repertoire (n = 86) of wild foraged mushrooms. As before, small portions of the dried mushrooms were briefly mixed with 1 mL of PBS, and a 100 μL aliquot of the extract was immediately applied to the sample pad of the LFIA test strip. The line intensity was interpreted and recorded within 10 minutes and a digital image was also acquired for each test strip.

To confirm the presence or absence of α-AMA, LC-MS analysis was conducted on species known to contain amatoxins (*A*. *bisporigera*, *A*. *ocreata*, *A*. *phalloides*, *A*. *marmorata*, *Galerina marginat*a, and *Lepiota subincarnata*) and on four closely related species that were known to not contain amatoxins (*A*. *constricta*, *A*. *gemmata*, *A*. *muscaria*, *and A*. *pantherina)*. Extraction was performed using dried mushroom tissue extracted using methanol-water-0.01 M HCl (5:4:1, v/v/v) at a ratio of 100 mg of dried mushroom to 1 mL of extraction buffer. The tissue was incubated with shaking for 30 minutes at room temperature, and then centrifuged at 10,000 x g for 10 mins. The supernatant was removed and analyzed by LC-MS/MS/MS for α-AMA and, for one specimen, by ultra-high pressure liquid chromatography-high resolution accurate mass spectrometry (UHPLC-HRAMS) for phalloidin and phallacidin.

Mushroom extracts were analyzed for α-AMA according to a previously described LC-MS/MS/MS method with slight modifications [36]. In brief, the samples were analyzed using a Thermo Velos Pro linear ion trap mass spectrometer interfaced with a Dionex Ultimate 3000 UHPLC system (Thermo, San Jose, CA, USA). The HPLC was fitted with a 2.1 x 50 mm, 1.8 μm Agilent Zorbax SB-C18 column (Agilent, Santa Clara, CA, USA). Mobile phases were water (A) and acetonitrile (B), each containing 0.1% formic acid. Gradient elution was used, initially set at 5% B, held for 1.5 minutes, then increased to 30% B at 7 minutes and then 90% B at 9 minutes. At 9.1 minutes the solvent composition was set back to 5% B and the column re-equilibrated for 6 minutes. The column flow rate was 0.35 mL/min and the injection volume was 2.0 μL. Mass spectrometer ionization conditions and ion transitions were as per the previously published method [36]. Results were reported as positive if the retention time on the total ion chromatogram and the MS fragmentation aligned with the standard solution of α-AMA.

One extract (*A*. *marmorata*) was analyzed for phalloidin and phallacidin using a Thermo Q-Exactive high resolution accurate mass spectrometer (Thermo) interfaced to a Dionex Ultimate 3000 UHPLC. The HPLC was fitted with a 2.1 x 100 mm, 1.7 μm Agilent Eclipse Plus C-18 column (Agilent). Mobile phases were water (A) and acetonitrile (B), each containing 0.1% formic acid. Gradient elution was used, initially set at 1% B, held for 1.5 minutes, then increased to 98% B at 9.5 minutes. It was held at 90% B until 13.5 minutes and then set back to 1% B and re-equilibrated for 4 minutes. The flow rate was 0.35 mL/min and injection volume was 20 μL. Positive electrospray ionization was used. Parallel reaction monitoring was used to provide three scan functions. The first collected full scan spectra from *m/z* 75–1125 with 70,000 mass resolution at *m/z* 200. The second was used to collect MS/MS fragment ion spectra of *m/z* 789, the [M+H]^+^ ion for phalloidin. The third collected MS/MS fragment ion spectra of *m/z* 847, the [M+H]^+^ ion for phallacidin. Both MS/MS scan functions used 17,500 mass resolution at *m/z* 200 and stepped collision energy at 35, 45, and 55 eV. Results were reported as positive if the retention time on the total ion chromatogram and the MS fragmentation aligned with the standard solution of phalloidin or phallacidin.

The PBS-based extracts obtained from the *A*. *marmorata* and *A*. *bisporigera* samples were diluted 1000-fold and 100,000-fold in PBS and analyzed by LFIA. This was performed in order to evaluate if the diluted sample would dilute out the detection of the phallotoxins and amatoxins, respectively.

## Results and discussion

**Fig 2:**
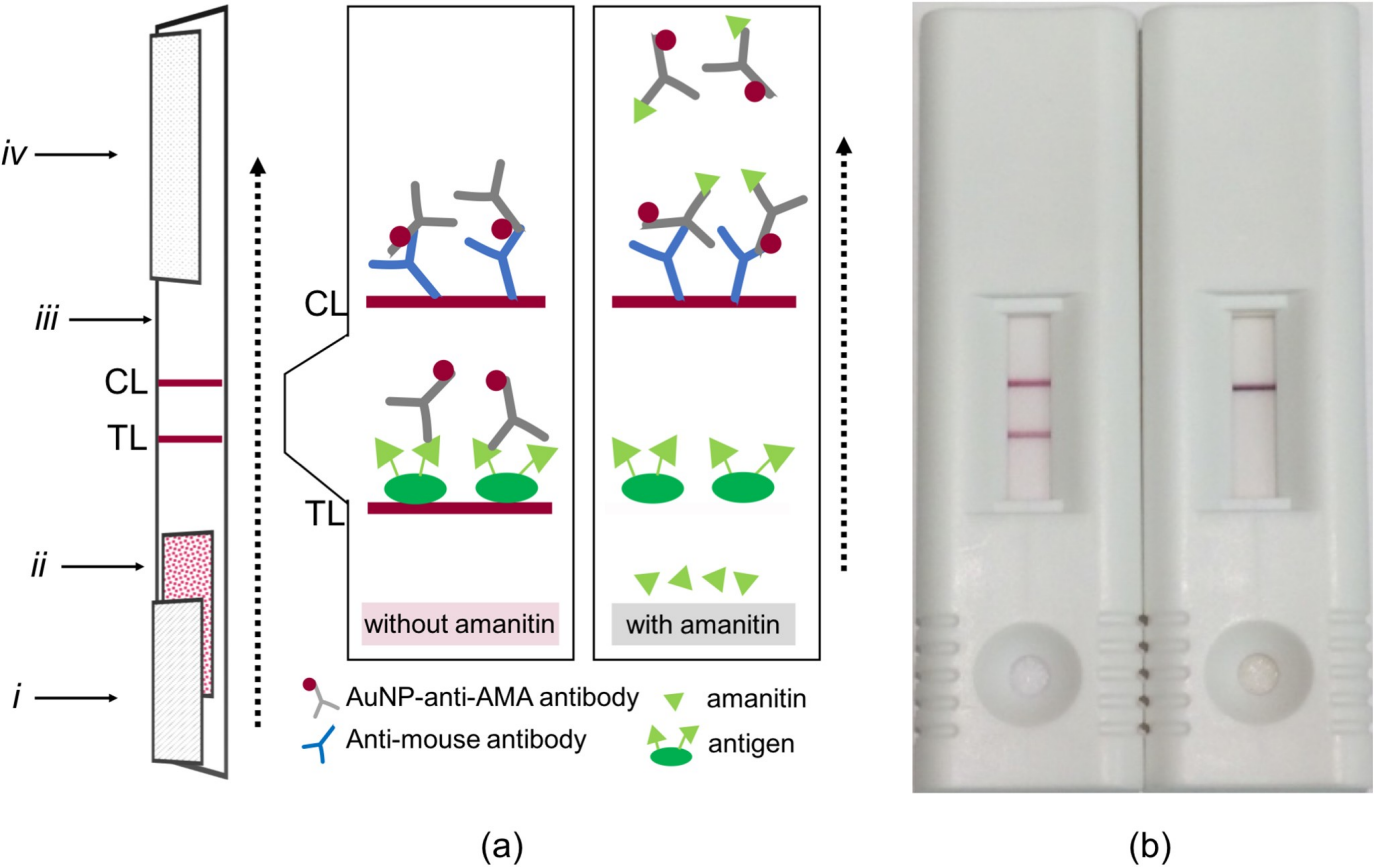
Depictions of the test strips used in this study. (a) Schematic diagram of the lateral flow strip along with a diagram of the reagents on the control line (CL) and test line (TL). (b) A view of the strips when used in a cassette. The left cassette is an example of a sample without amatoxins (negative) and the right cassette is an example of a sample with amatoxins (positive). (*i*) sample pad, (*ii*) conjugate pad, (*iii*) nitrocellulose membrane, (*iv*) wicking pad, and the arrow indicates the flow direction.

The LFIA for amatoxin detection was developed and performed in a competitive inhibition assay format. A schematic of the test strip, along with an example of a negative and positive test, is shown in Fig 2. The sample to be tested is added to the sample pad, which interacts with and rehydrates the gold-labeled antibody pre-loaded on the conjugate pad. A competitive assay works such that if amatoxins are present at a high enough concentration in the sample, the antibodies will bind to the amatoxins, thus not allowing the antibodies to bind to the antigen immobilized at the test line, which results in no visible line. As a control to ensure the test is valid, the gold-labeled antibodies will bind to the anti-mouse antibody immobilized at the control line, thus producing a visible control line.

### Optimal concentrations of antibody-gold conjugation and immobilized capture reagents

The optimal conditions required to stabilize the colloidal gold particles with mAb AMA9G3 antibody protein were to perform the conjugation at a pH of 8 or greater and using 2 μg/mL of antibody or greater. Since the assay would be a competitive format wherein the toxin is meant to displace the antibody binding, we used this lowest acceptable antibody loading of 2 μg/mL.

**Fig 3:**
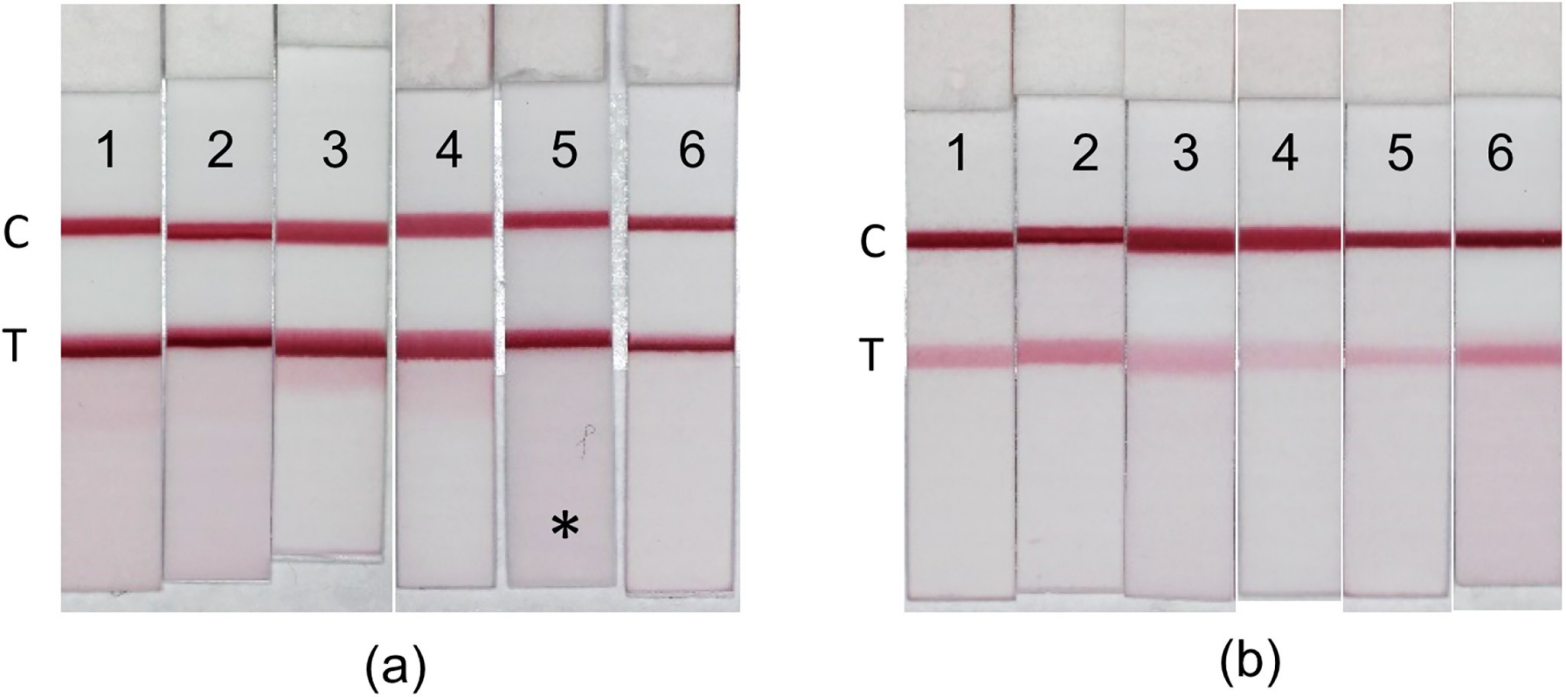
Visual representation of the lateral flow immunoassay (LFIA) half strips. Test (T) line coating antigens were (a) LB-AMA BSA and (b) PERI-AMA-BSA immobilized onto six different nitrocellulose membrane types: (1) MDI 150, (2) FF120, (3) FF80, (4) MDI 90, (5) CN95, and (6) CN140. (*) designates the preferred membrane used in the remaining experiments.

Preliminary testing established that immobilizing goat anti-mouse IgG using a solution at 1.0 mg/mL was sufficient for a visible control line. For the test line, two conjugates were tested in a half strip format, PERI-AMA-BSA coated at 11 mg/mL and LB-AMA-BSA coated at 1 mg/mL, both on 6 different nitrocellulose membrane types. The line intensity for the test line coated with the LB-AMA-BSA antigen was considerably higher than the test lines coated with PERI-AMA-BSA (Fig 3). Therefore, the LB-AMA-BSA antigen was the preferred coating antigen used for the remaining tests. Based on line morphology and membrane background, CN95 was the preferred membrane and was used for the remaining experiments. In addition, because there was evidence of a darker leading edge on the test line, to make the coloration appear more uniformly distributed, the antigen coating concentration was reduced down to 0.5 mg/mL for the full strip production used for the remaining experiments.

### Analytical detection of α-, β-, and γ-amanitin by lateral flow immunoassay (LFIA)

**Fig 4:**
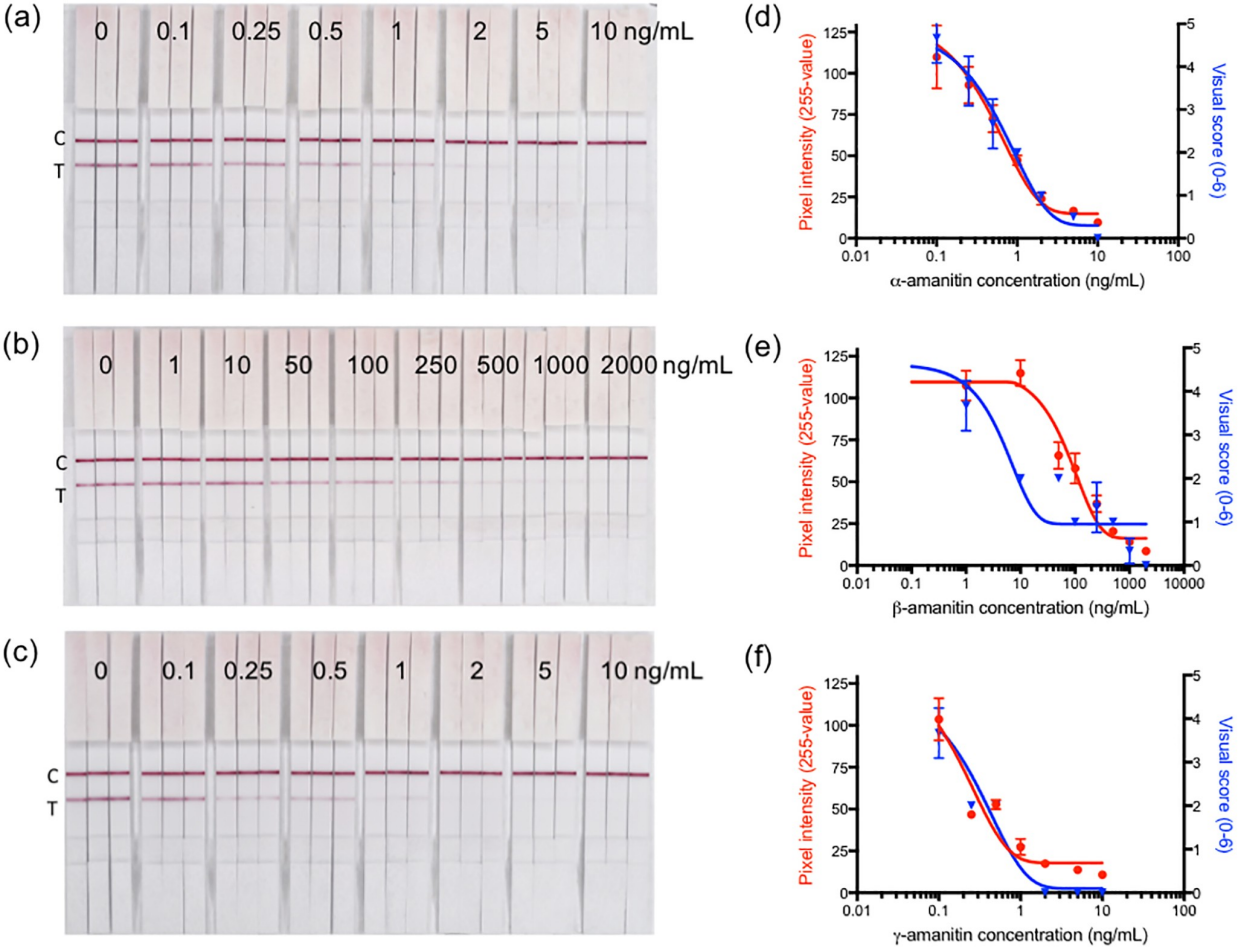
LFIA detection of amatoxins. Standard calibration curves of (a) α-amanitin, (b) β-amanitin, and (c) γ-amanitin determined by lateral flow immunoassay (LFIA). The images on the left (a-c) are the test strips. The graphs to the right (d-f) are the test line pixel values (red circles) and visual score values (blue triangles) from the corresponding image (a-c) expressed as a mean ± standard error, for three separate strips.

To generate a standard calibration curve of the LFIA for the three most common amanitins, solutions of different concentrations of α-AMA, β-AMA, and γ-AMA in PBS were assessed (Fig 4). Digitally-acquired pixel values correlated extremely well with the subjective visual scoring on a scale of 0–6 for α-AMA and γ-AMA (Fig 4a and 4c), and moderately so for β-AMA (Fig 4b). For the β-AMA plot, the misalignment seems to be driven by the visual score data point (blue triangle) at 10 ng/mL, while the remaining visual score points trend with the pixel values, and thus the misalignment is likely due to the subjective scoring by eye. In order to remove ambiguity in reporting results, we defined the analytical cut-off value as the concentration in which the test line is completely absent due to the competitive inhibition by the toxin in a sample solution competing with the gold-labeled antibody. The cut-off value for α-AMA and γ-AMA was 10 ng/mL (0.1 μg toxin/g mushroom) and the cut-off for β-AMA was 2000 ng/mL. These results corroborate what we observed when using this mAb in an ELISA format wherein mAb AMA9G3 exhibited a lower IC_50_ for α-AMA and γ-AMA than for β-AMA [32]. Based on the digitized pixel values (shown as the red lines in Fig 4), the limit of detection (LOD; defined as three times the standard deviation of a sample without amanitin) is 0.3 ng/mL for α-AMA and γ-AMA and 30 ng/mL for β-AMA. The LFIA’s cut-off value for α-AMA is comparable to the LOD for LC-MS methods used for α-AMA detection in mushroom analysis [24–26].

For competitive LFIAs, it is often hard to discern if the line is simply fainter (and therefore partially inhibited) due to the presence of toxin or possibly from lighting conditions, age of the strips, time of reading, or other unknown or unanticipated variables. This uncertainty is observed in the only other published LFIA for amatoxins, wherein the authors note that at 2, 10 and 20 ng/mL of α-AMA the line is still present, although decreased visual intensity than from the “no toxin” test line [33]. In our experience, if our LFIA were read hours (or even days) after development (instead of the suggested 10 mins), a faint line would appear for the standards containing 5 ng/mL of α-AMA or less, yet no line appears for 10 ng/mL of α-AMA standard. Thus, defining the cut-off value for this LFIA at 10 ng/mL for α-AMA gives greater confidence, and less ambiguity in the interpretation of consistent results.

### Analytical selectivity of the LFIA

To ensure that the LFIA is accurate and selective for amatoxin detection, chemical standards of closely related compounds and other cyclic peptides were tested for cross-reactivity (Table 1). No detection was observed for mushroom toxins psilocybin, muscimol, and ibotenic acid, nor for cyclic peptides microcystin-LR or nodularin. In contrast, α-AMA and γ-AMA have similar LODs, which are lower than the LOD for β-AMA. The cross-reactivity for β-AMA by LFIA is 0.5%. Although this is a small value, given the large quantity of β-AMA in known mushroom specimens (approximately 1–2 mg/g (dried) [37]), it would be detectable in a typical extraction (1 mL per approximately 100 mg of dried tissue) and detectable at up to a 100-fold dilution of that extract.

**Table 1:** LFIA test results for pure chemical toxin standards of chemicals from associated mushrooms or are other peptide toxins.

Toxin	Concentration tested (μg/mL)	LFIA result (n = 3)	Cross Reactivity^a^ (%)
**α-amanitin**	0.01	+ + +	100
**γ-amanitin**	0.01	+ + +	100
**β-amanitin**	2	+ + +	0.5
**phalloidin**	200	+ + +	0.005
	20	– – –	
	2	– – –	
**phallacidin**	200	+ + +	0.005^b^
	20	+ – –	
	2	– – –	
**psilocybin**	100	– – –	nd^c^
**microcystin-LR**	20	– – –	nd
**nodularin**	10	– – –	nd
**ibotenic acid**	200	– – –	nd
**muscimol**	200	– – –	nd

^a^ Cross-reactivity (%) = ([cut-off value of α-AMA] / [cut-off value of the test inhibitor] x 100.

^b^ value estimated from the LFIA reading with the highest majority

^c^ nd = not determined because the analyte was not detected.

The LFIA cross-reacts with the phallotoxins (phallacidin and phalloidin) at 0.005%, or a concentration of 200 μg/mL. This was not seen in our previously developed ELISA using the same mAb AMA9G3 [32], because the highest concentrations tested for these analytes in our earlier study were lower than 2 μg/mL. These phallotoxins are often found in *Amanita* species at approximately 1–2 mg/g of dried mushroom [17, 37], which are at comparable concentrations to the amatoxins. At the current extraction volume described here, a positive result could be due to the phallotoxins.

There are numerous other chemicals within the classes of amatoxins and phallotoxins for which chemical standards are not currently commercially available, such as ε-amanitin, amanin, amaninamide, amanullin, amanullinic acid, and proamanullin, as well as phallacin, phallisin, phalloin, prophalloin, and phallisacin. While they could not be tested for cross-reactivity in this assay, their concentrations and distributions in mushrooms are also not well-described in the literature. Nonetheless, since an antibody binds molecules based on molecular shape and not exact chemical composition, it is conceivable that any of these molecules might be present in a sample. Furthermore, without standards at this time, those samples cannot be definitively confirmed by techniques, such as LC-MS. And thus, the LFIA might produce a positive result although instrumental LC-MS methods cannot validate it at this time.

### Shelf-life testing of the LFIA

**Fig 5:**
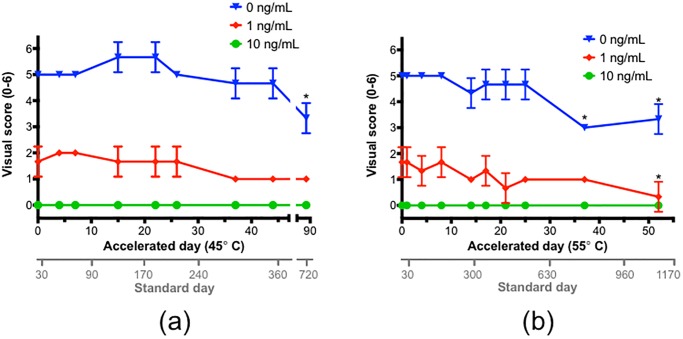
Shelf-life testing of the LFIA stored at (a) 45 °C and (b) 55 °C. Minimal loss of signal was observed over the course of 25 days for those tested at 55 °C and over the course of 44 days for those tested at 45 °C. The LFIA performance was tested using 3 different concentrations of α-AMA (0, 1, and 10 ng/mL) in PBS.

The shelf-life of a product can be estimated by performing an accelerated stability study. Each day that a product is held at an elevated temperature equates to a presumed stability for an equivalent duration of standard days at room temperature [35]. We stored test strips at 45 °C and at 55 °C, for up to 87 days and 52 days respectively. Sets of test strips were removed periodically and tested using three different concentrations of α-AMA (0, 1, and 10 ng/mL) in PBS. Overall, no statistically significant loss in signal was observed for the first 44 accelerated days for the strips held at 45 °C and for the first 25 accelerated days for the strips held at 55 °C (Fig 5). The stability at these accelerated days equate to a minimum shelf-life of approximately 360 standard days (1 year) and 540 standard days (1.5 years), respectively.

Testing of different α-AMA concentrations provided us a way to identify if sensitivity was impacted along with overall signal intensity. The consistency of the signal intensity over time was observed in the experiments when 0 ng/mL of α-AMA (only PBS) was used. A decrease in signal intensity was observed using strips from accelerated day 87 held at 45 °C and at accelerated days 37 and 52 for the strips held at 55 °C (Fig 5). The study was not maintained longer than the latter of those accelerated time points. In addition, the signal intensity also dropped statistically significantly (compared to the signal intensity from day 0) for one other time point, accelerated day 52, for the strips held at 55 °C and tested with 1 ng/mL of α-AMA. These observed drops in signal intensity were no more than 2 points on the 6-point visual score. No signal was ever observed for the strips tested with 10 ng/mL of α-AMA, at either temperature, which was expected since this amount of α-AMA should eliminate the presence of the test line completely.

Although signal intensity decreased over time in this accelerated stability study, when the LFIA was exposed to elevated temperatures, the entire signal was not completely diminished. Thus, the LFIA still produced reliable qualitative results for all the conditions tested. The decrease in signal intensity after 1–1.5 standard years could serve as an internal product monitor to know when a batch of strips may need to be replaced. Furthermore, a year or more shelf-life is desirable for a product like this in which the appearance of mushrooms and their related poisonings typically occur seasonally each year.

### Detection of amatoxins in foraged wild mushrooms

We tested 110 foraged mushrooms, comprised of 96 different species (Table 2) for the presence or absence of amatoxins. The mushrooms were dried specimens collected anywhere between 1 day to 20 years prior to performing this testing. Most of the mushrooms were identified to species by expert mycologists using morphology. For some of the mushrooms that are difficult to differentiate beyond the genus level, species identification was confirmed by DNA sequencing of the internal transcribed spacer (ITS) region [38, 39]. The DNA sequence of the ITS region was then BLAST searched in the NCBI database to assign a species based on the highest percent match.

**Table 2:** Species names and UC Herbarium codes for wild mushrooms sampled in this study and mentioned in Fig 6. A ‘Y” in the “ITS” (internal transcribed spacer) column indicates that the ITS region was sequenced and assigned a species based on the highest percent match when BLAST searched in the NCBI database. Bold text indicates those species that are known to contain amatoxins. Those marked with an * were subjected to chemical analysis by LC-MS.

No.	Species identification	UC Herbarium code	ITS	No.	Species identification	UC Herbarium code	ITS
1	*Agaricus californicus*	UC 2060385		56	*Gastroboletus turbinatus*	UC 1998587	
2	*Agaricus xanthodermus*	UC 2060384	Y	57	*Gastroboletus vividus*	UC 1860875	
3	*Agrocybe pediades*	UC 1998617	Y	58	*Gastroboletus vividus*	UC 1998577	
4	*Amanita augusta*	UC 2060350		59	*Gyromitra gigas*	UC 199122	
5	Amanita bisporigera*	UC 2060392		60	*Handkea subcretacea*	UC 1998567	
6	*Amanita calyptratoides*	UC 2060368	Y	61	*Hemimycena delectabilis*	UC 1998640	
7	Amanita constricta*	UC 2060356		62	*Homophron spadiceum*	UC 1999292	
8	Amanita gemmata*	UC 2060365		63	*Hypholoma fasciculare*	UC 1998522	
9	*Amanita magniverrucata*	UC 2060358		64	*Hypholoma fasciculare*	UC 1998517	
10	Amanita marmorata*	UC 2060363		65	*Ischnoderma resinosum*	UC 1998572	
11	Amanita muscaria*	UC 2060362	Y	66	*Kuehneromyces vernalis*	UC 1998746	
12	*Amanita muscaria*			67	*Lactarius deliciosus*	UC 1860851	Y
13	*Amanita novinupta*	UC 2060389		68	*Leccinum manzanitae*	UC 1998720	
14	Amanita ocreata*	UC 2060355		69	*Lepiota aspera*	UC 2060096	
15	***Amanita ocreata***			70	*Lepiota castaneidisca*	UC 1999327	
16	*Amanita pachycolea*	UC 2060372		71	*Lepiota cf*. *cristata*	UC 2060101	Y
17	Amanita pantherina*	UC 2060395		72	*Lepiota flammeatincta*	UC 2060193	
18	Amanita phalloides*	UC 2060369		73	*Lepiota luteophylla*	UC 2060057	
19	***Amanita phalloides***			74	*Lepiota rhodophylla*	UC 2060056	
20	*Amanita protecta*	UC 2060370		75	*Lepiota sequoiarum*	UC 2050032	
21	*Amanita sylvicola*	UC 2060375		76	*Lepiota spheniscispora*	UC 2060100	
22	*Amanita velosa*	UC 2060361		77	Lepiota subincarnata*	UC 2060054	
23	*Boletus appendiculatus* s.l.	UC 1998735		78	***Lepiota subincarnata***	UC 2060095	
24	*Boletus edulis*	UC 2060353		79	*Lepiota* sp. sect. *Stenosporae*	UC 2060030	
25	*Boletus fibrillosus*	UC 1998721		80	*Leucoagaricus erythrophaeus*	UC 1999375	
26	*Boletus fibrillosus*	UC 1998574		81	*Leucocoprinus brebissonii*	UC 2060403	
27	*Boletus rex-veris*	UC 1998729		82	*Morchella* sp.	UC 1999063	
28	*Boletus rubripes*	UC 1861056	Y	83	*Melanoleuca angelesiana*	UC 1998764	
29	*Butyriboletus abieticola*	UC 1998732		84	*Melanoleuca melaleuca*	UC 1998913	
30	*Calbovista subsculpta*	UC 1998863		85	*Melanoleuca robertiana*	UC 1998614	Y
31	*Calbovista subsculpta*	UC 1998751		86	*Mycena nivicola*	UC 1998796	
32	*Caloboletus frustosus*	UC 1860877	Y	87	*Myxomphalia maura*	UC 1999137	
33	*Caloboletus roseipes*	UC 1860855	Y	88	*Nolanea verna*	UC 1998642	Y
34	*Caloscypha fulgens*	UC 1999117		89	*Peziza repanda*	UC 1998869	
35	*Caloscypha fulgens*	UC 1998915		90	*Pholiotina gracilenta*		Y
36	*Cantharellus californicus*	UC 2060357		91	*Pholiotina utricystidiata*		Y
37	*Chroogomphus albipes*	UC 1861050		92	*Phyllotopsis nidulans*	UC 1999138	
38	*Chroogomphus pseudovinicolor*	UC 1861026		93	*Phyllotopsis nidulans*	UC 1998641	
39	*Chrysomphalina aurantiaca*	UC 1860175		94	*Plicaria endocarpoides*	UC 1861196	
40	*Citocybe glacialis*	UC 1998610		95	*Psathyrella piluliformis*	UC 1998613	
41	*Clitocybe nuda*	UC 1998524		96	*Rhodocollybia maculata*	UC 2060373	
42	*Clitocybe squamulosa*	UC 1998763		97	*Rhodophana nitellina*	UC 1998616	
43	*Clitocybe* sp.	UC 1999055		98	*Rubroboletus haematinus*	UC 1861053	
44	*Connopus acervatus*	UC 1999132		99	*Russula favrei*	UC 1860891	Y
45	*Cortinarius cephalixus*	UC 1998661	Y	100	*Sarcosphaera* cf. *coronaria*	UC 1998862	
46	*Cortinarius cyanites*	UC 1999129		101	*Spongiporus leucospongia*	UC 1860874	Y
**No.**	**Species identification**	**UC Herbarium code**	**ITS**	**No.**	**Species identification**	**UC Herbarium code**	**ITS**
47	*Cortinarius gentilis*	UC 1999046		102	*Spongiporus leucospongia*	UC 1860895	Y
48	*Cortinarius rubicundulus*	UC 1999317		103	*Suillellus amygdalinus*	UC 1998733	
49	*Cortinarius subalpinus*	UC 1998860		104	*Tapinella atrotomentosa*	UC 1999002	
50	*Cortinarius* subgenus *seriocybe*	UC 1998569	Y	105	*Tricholomopsis rutilans*	UC 1998579	Y
51	*Cortinarius* sp.	UC 1999036		106	*Tricholomopsis rutilans*	UC 1860853	Y
52	*Entoloma trachyspermum*	UC 1999311		107	*Volvariella volvacea*	UC 2060349	
53	*Fomitopsis pinicola*	UC 1998908		108	*Xerocomus subtomentosus*	UC 1998765	
54	Galerina marginata*	UC 2060366	Y	109	*Xeromphalina campanella*	UC 1998761	
55	*Galerina sideroides*		Y	110	*Xeromphalina campanella*	UC 1998609	

**Fig 6:**
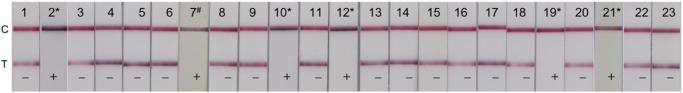
LFIA results from mushroom extracts. The mushrooms are as follows: 1) *Amanita augusta*, 2) *A*. bisporigera*, 3) *A*. *calyptratoides*, 4) *A*. *constricta*, 5) *A*. *gemmata*, 6) *A*. *magniverrucata*, 7) *A*. *marmorata*^#^, 8) *A*. *muscaria*, 9) *A*. *novinupta*, 10) *A*. ocreata*, 11) *A*. *pantherina*, 12) *A*. phalloides*, 13) *A*. *protecta*, 14) *A*. *velosa*, 15) *Agaricus californicus*, 16) *Ag*. *xanthodermus*, 17) *Boletus edulis*, 18) *Cantharellus californicus*, 19) Galerina marginata*, 20) *G*. *sideroides*, 21) Lepiota subincarnata*, 22) *Pholiotina utricystidiata*, and 23) *Volvariella volvacea*. Those marked with an * were confirmed by LC-MS analysis to contain α-AMA, and the sample marked with a ^#^ was confirmed by LC-MS analysis to contain phallotoxins. 74 additional mushroom species tested were negative by LFIA. Names of all mushrooms tested are provided in Table 2.

For amatoxin detection, a small (<200 mg) piece of dried mushroom was extracted into PBS and swirled for a few seconds. An aliquot (100 μL) of the extract was then placed onto the sample pad of the LFIA. The results were clearly visible by 5 mins, but for experimental consistency strips were read at 10 mins. The specimens that tested positive by LFIA, resulting in a complete absence of the test line, were *Amanita bisporigera*, *A*. *marmorata*, *A*. *ocreata* (both specimens), *A*. *phalloides* (both specimens), *Galerina marginata*, and *Lepiota subincarnata* (both specimens) (a subset of strips is shown in Fig 6). All of these specimens (at least one specimen from each species) were confirmed for the presence of α-AMA by LC-MS analysis (S1 Table) except *A*. *marmorata*. The other 90 mushroom species sampled by LFIA were negative for amatoxins (a subset of strips is shown in Fig 6). Four of the other *Amanita* specimens (*A*. *constricta*, *A*. *gemmata*, *A*. *muscaria*, and *A*. *pantherina*) were confirmed negative for α-AMA by LC-MS (S1 Table).

Although, in this study, one *A*. *marmorata* specimen, that was positive by LFIA and did not contain detectable α-AMA by LC-MS, the presence of phallotoxins were confirmed by LC-MS analysis (S2 Table). This result demonstrates that this specimen does make cyclopeptide toxins and thus possesses the cycloamanide gene family [11, 15, 40]. Variability in toxin production (i.e., some specimens within this species has produced detectable amounts of amatoxins and/or phallotoxins) has been observed in *A*. *bisporigera*, *A*. *marmorata*, and *A*. *suballiacea* [11, 41]. Upon further evaluation, the 1000-fold dilution of extracts from *A*. *marmorata* and *A*. *bisporigera* were also positive by LFIA. The 100,000-fold extracts from both specimens tested negative by LFIA, which is expected as this would dilute the amatoxins to below detectable amounts. In theory, given the low cross-reactivity with phallotoxins, a 10-fold dilution of the extract would be sufficient to dilute the phallotoxins to non-detectable amounts. However, antibody-based detection is unique in that all of the amatoxins and phallotoxins (even those for which analytical standards aren’t available) bind cumulatively and present as a single result—the simple presence or absence of a line. While the LFIA does minimally (0.005%) cross-react with phallotoxins, we cannot exclude the possibility that a false positive result for *A*. *marmorata* is due to phallotoxins alone. A complete set of chemical standards are needed to establish a conclusion. Thus, the LFIA is a useful screening tool, which is identifying species producing cyclopeptides. Further research with appropriate chemical standards would help to provide definitive experimental evidence to validate which particular cyclopeptides are present.

To our knowledge, this is the first demonstration of a LFIA for the detection of amatoxins in authentic amatoxin-containing mushroom samples. The speed of extraction and detection (~10 mins), along with the accuracy of identifying amatoxin-containing mushroom species obtained by this LFIA test is remarkably faster than current antibody-based or LC-MS methods, which take a minimum of an hour to obtain a result [24–26, 31, 42]. A previously reported LFIA for amatoxins, testing amanitin-spiked mushroom samples, utilized a 90 minute extraction procedure using a methanol-water solution and the extracts required dilution in order to reduce matrix effects [33]. Since the matrix effects in their assay were likely due to the presence of methanol, sample extraction and dilution could probably be simplified using the extraction procedure described in our work.

For mushroom analysis, LC-MS, ELISA, and our LFIA method exhibit comparable analytical LOD in the ng/mL range [24–26, 31, 32, 34, 42]. In addition, most of the amatoxin-containing specimens contain 2–4 mg/g of total amatoxins per dried (cap) tissue [24, 37, 43–45]. Together this means that our extracts of dried amatoxin-containing mushrooms can undergo a 10,000-fold dilution and still be detectable. For LFIA detection along with the extraction method described in this paper, the extraction volumes that could be used while still detecting amatoxins from approximately 10 mg of dried mushroom cap tissue can range from 1 mL to 1 L. In addition, our LIFA has worked on fresh specimens extracted using the same rapid protocol. Fresh specimens contain around 90% water, and therefore toxins are 10-fold more concentrated in a dried specimen. Nonetheless, this large range of suitable extraction weights and volumes is desirable for field testing where precise measurements can be avoided.

Rapid amatoxin detection can be used to augment existing techniques used by mycologists when describing new species of mushrooms. To date, it is reported that over 10,000 mushroom species have been named and fully described, although this is likely only 1% of the total species of fungi in the world [46]. This test would be particularly helpful when distinguishing mushrooms with relatively few diagnostic features, such as *Galerina* or *Conocybe* species. The misidentification of mushrooms by conventional mycological evaluation (i.e., spore print, habitat, morphological characteristics) can lead to unintended detrimental outcomes. For instance, this LFIA test would be especially useful when collecting *Amanita* species, of which there are choice edibles (e.g., *A*. *hemibapha* and *A*. *princeps* in Southeast Asia, *A*. *velosa* in the USA) as well as deadly poisonous amatoxin-containing species [19]. A tool like LFIA could help alleviate confusion. For those with scientific resources, as a rapid chemical test, this LFIA could be paired with other technologies using DNA analysis [47]. Furthermore, toxin production may be evident in future sample identifications due to improved analytical technologies and interest.

Of the medical cases referred to the US Poison Control Centers, greater than 90% of the time the species of mushroom is unknown [48]. If a mushroom was available, most health care facilities would typically request the assistance of an expert mycologist. However, the mushroom may not be recognizable or retain its prominent characteristics needed to determine if it is a species that contains amatoxins. The LFIA test could be a valuable tool in health care settings to aid clinicians in identifying potential amatoxin poisonings.

This tool is not intended to determine edibility as there are numerous other toxins that can be present for which this test does not detect. For instance, *A*. *muscaria* contains hallucinogenic compounds (i.e., muscimol and ibotenic acid), while the *Agaricus* species tested contain unknown gastrointestinal irritants. None of these individual compounds or mushroom species cross-reacted with this assay, and therefore would not protect a person from becoming ill.

## Conclusions

This LFIA is a simple tool that detects amatoxins and does not require the use of harmful chemicals. The extraction of the mushroom tissue is performed in an aqueous buffer solution and is completed in less than a minute. Compared to ELISA formats, this LFIA has all of the immunoreagents pre-embedded in the design such that no additional reagents are needed at the time of testing aside from the sample extract. In addition, unlike both ELISA and LC-MS methods, the LFIA is a single step procedure from the point of sample addition and requires no washing steps. The total incubation time is 10 minutes and the result is simply identified by the presence or absence of the test line, without the need for specialized equipment. Furthermore, samples can be run simultaneously, whereas with LC-MS methods, each sample is run sequentially. This LFIA is a simple, sensitive, selective, portable, rapid, and accurate tool to detect amatoxins, which can aid in mushroom identification.

## Supporting information

S1 Table Total ion chromatograms (top) and mass spectrum (bottom) from the LC-MS analysis of mushroom extracts for the presence of α-amanitin. (DOCX)

S2 Table Total ion chromatograms (top) and mass spectrum (bottom) from the LC-MS analysis of the *A*. *marmorata* mushroom extract for the presence of phalloidin and phallacidin. (DOCX)
