# Supplementary material for: Lateral flow immunoassay (LFIA) for the detection of lethal amatoxins from mushrooms
Source: PLoS One. 2020 Apr 17;15(4):e0231781. doi: 10.1371/journal.pone.0231781 (PMC7164595; doi:10.1371/journal.pone.0231781)
Supplement: S1 Table — (DOCX) [file pone.0231781.s001.docx]

**Table S1. Total ion chromatograms (top) and mass spectrum (bottom) from the LC-MS analysis of mushroom extracts for the presence of α-amanitin.**

| **Species** | **Total ion chromatograms (top) and mass spectrum (bottom)** |
| --- | --- |
| *Amanita bisporigera* | 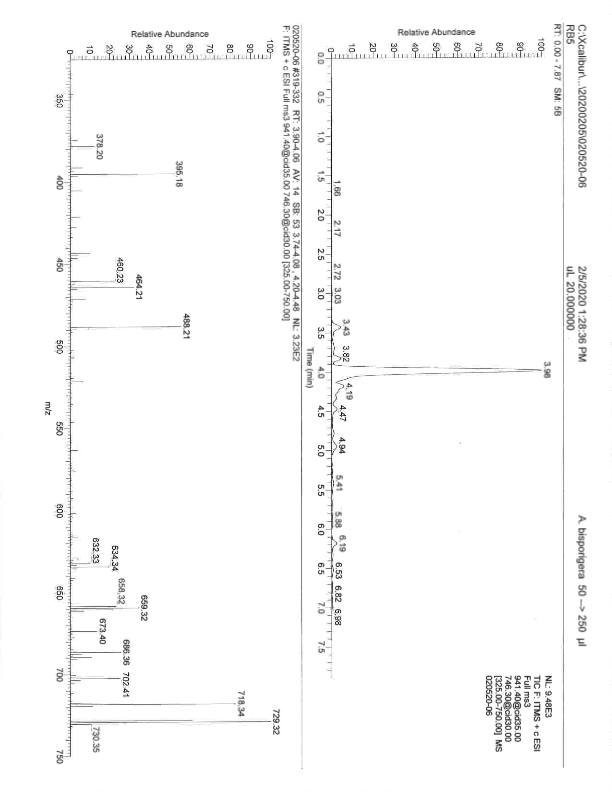 |
| *Amanita constricta* | 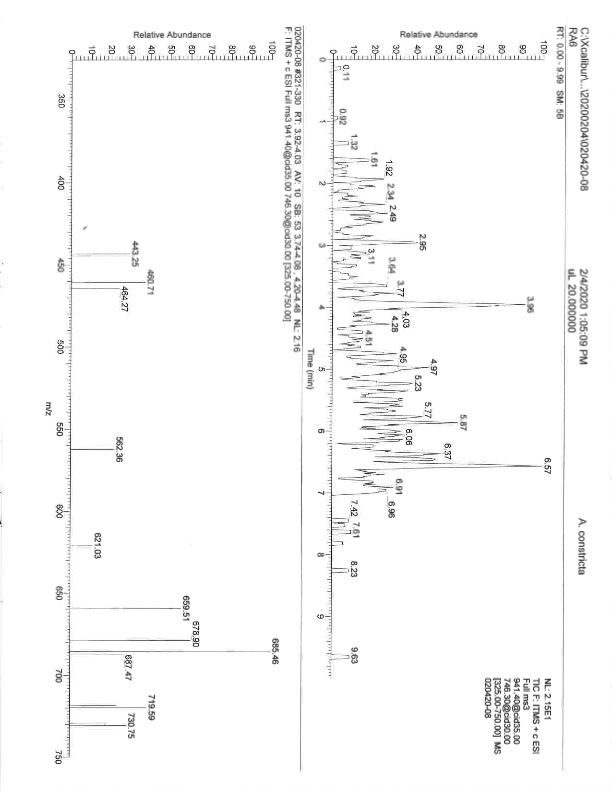 |
| *Amanita gemmata* | 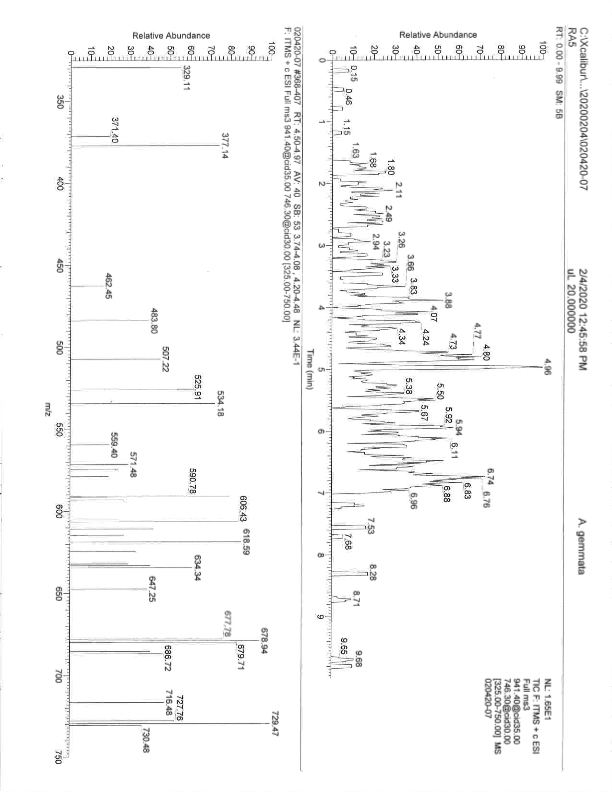 |
| *Amanita marmorata* | 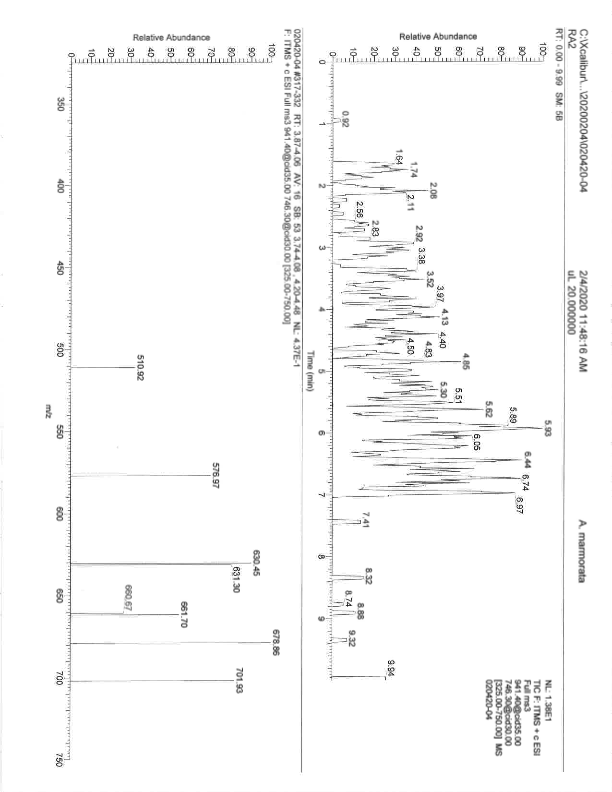 |
| *Amanita muscaria* | 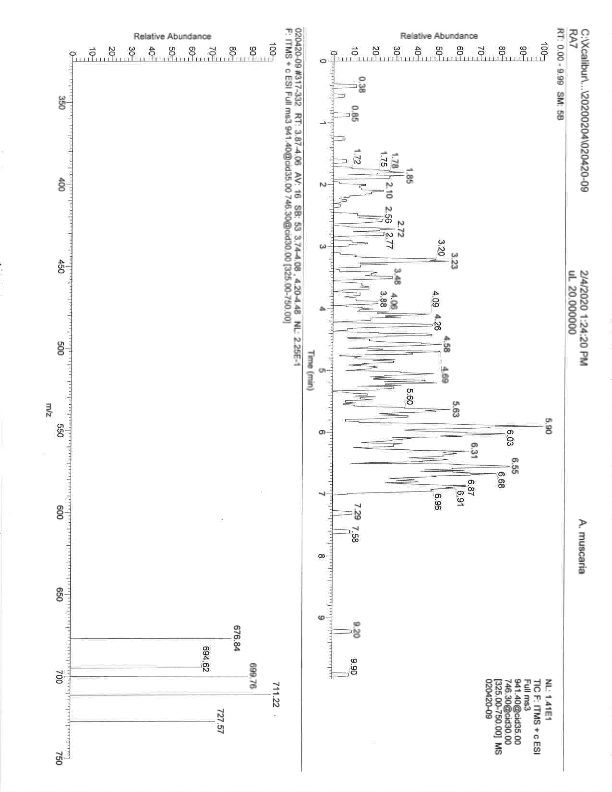 |
| *Amanita ocreata* | 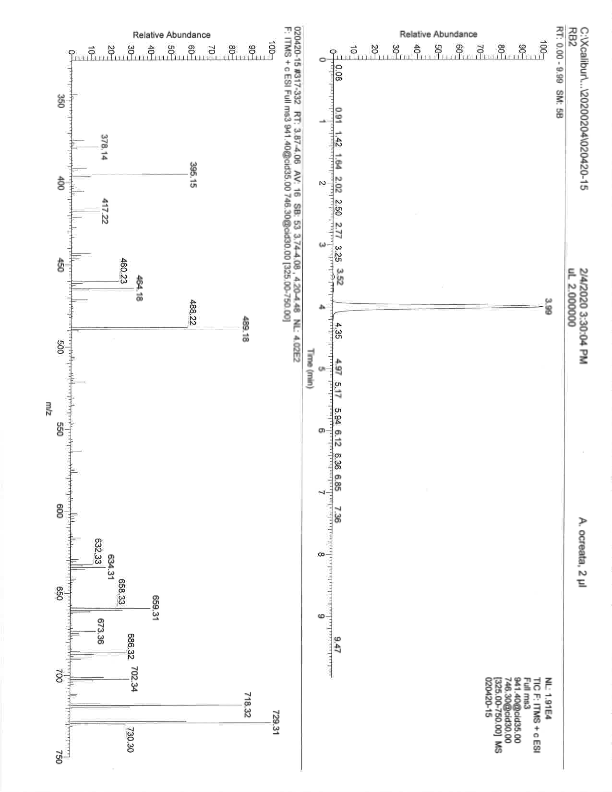 |
| *Amanita pantherina* | 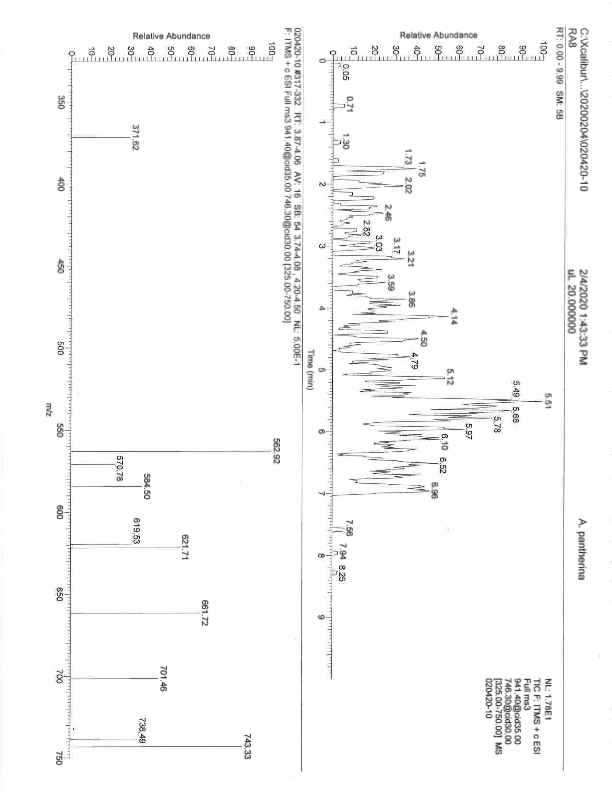 |
| *Amanita phalloides* | 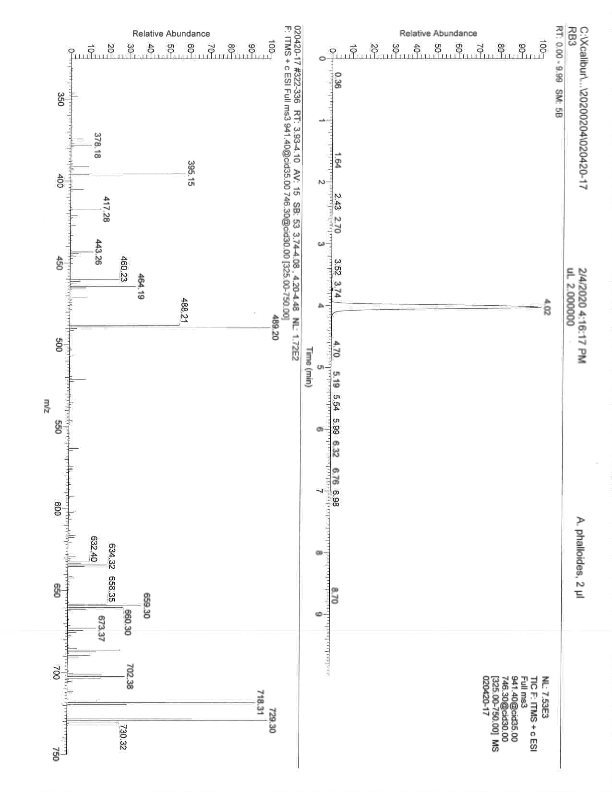 |
| *Lepiota subincarnata* | 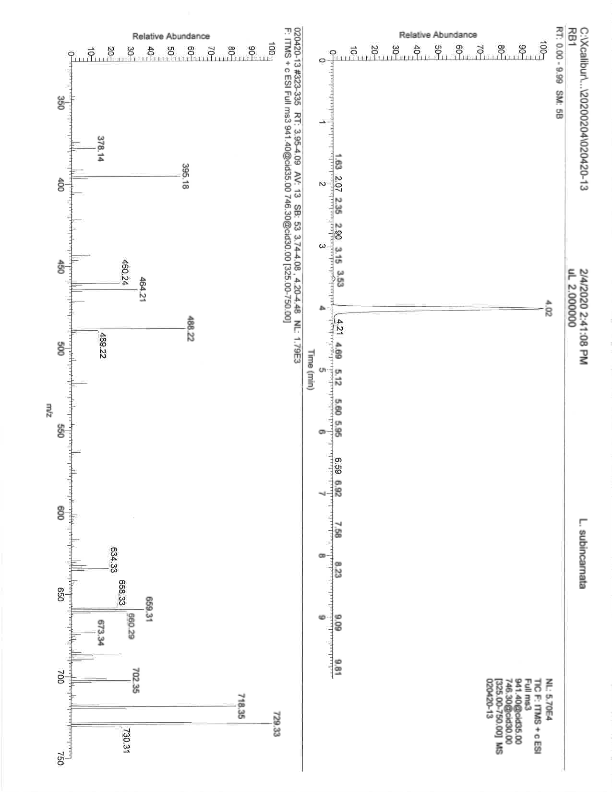 |
| *Galerina marginata* | 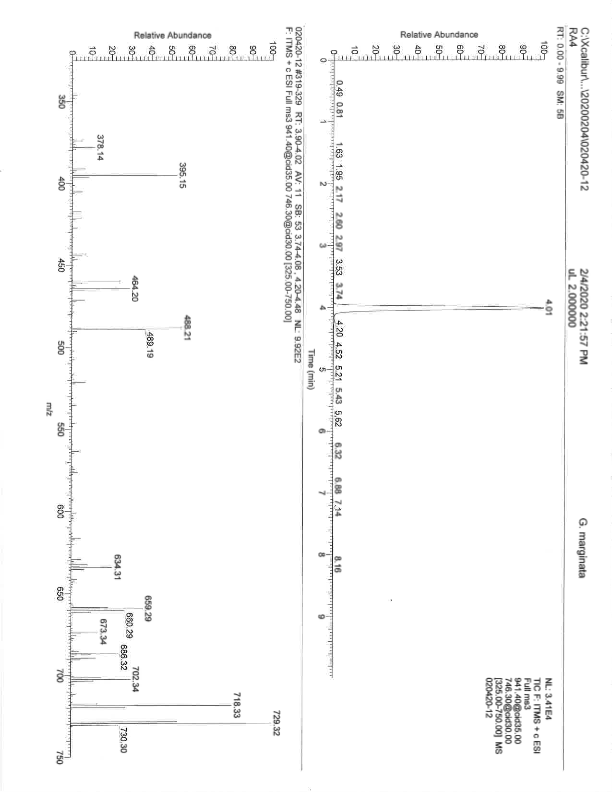 |
| α-amanitin  (reference standard) | 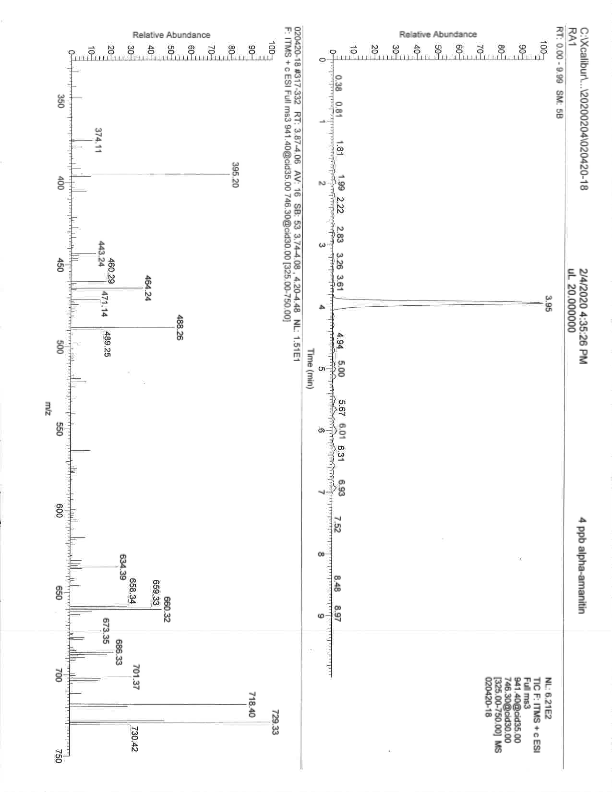 |
