# Supplementary material for: Lateral flow immunoassay (LFIA) for the detection of lethal amatoxins from mushrooms
Source: PLoS One. 2020 Apr 17;15(4):e0231781. doi: 10.1371/journal.pone.0231781 (PMC7164595; doi:10.1371/journal.pone.0231781)
Supplement: S2 Table — (DOCX) [file pone.0231781.s002.docx]

**Table S2. Total ion chromatograms (top) and mass spectrum (bottom) from the LC-MS analysis of the *A. marmorata* mushroom extract for the presence of phalloidin and phallacidin.**

| **Sample** | **Total ion chromatograms (top) and mass spectrum (bottom)** |
| --- | --- |
| *Amanita marmorata,* phalloidin | 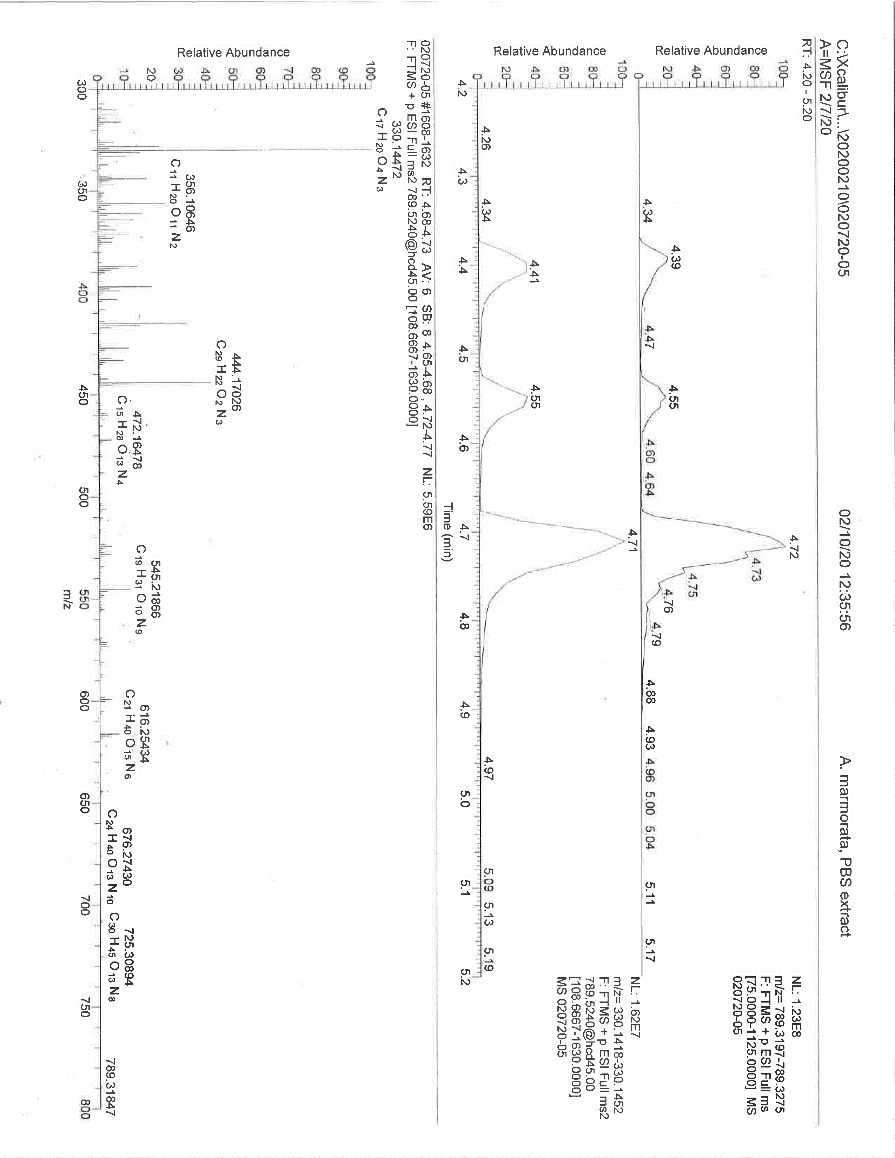 |
| *Amanita marmorata,* phallacidin | 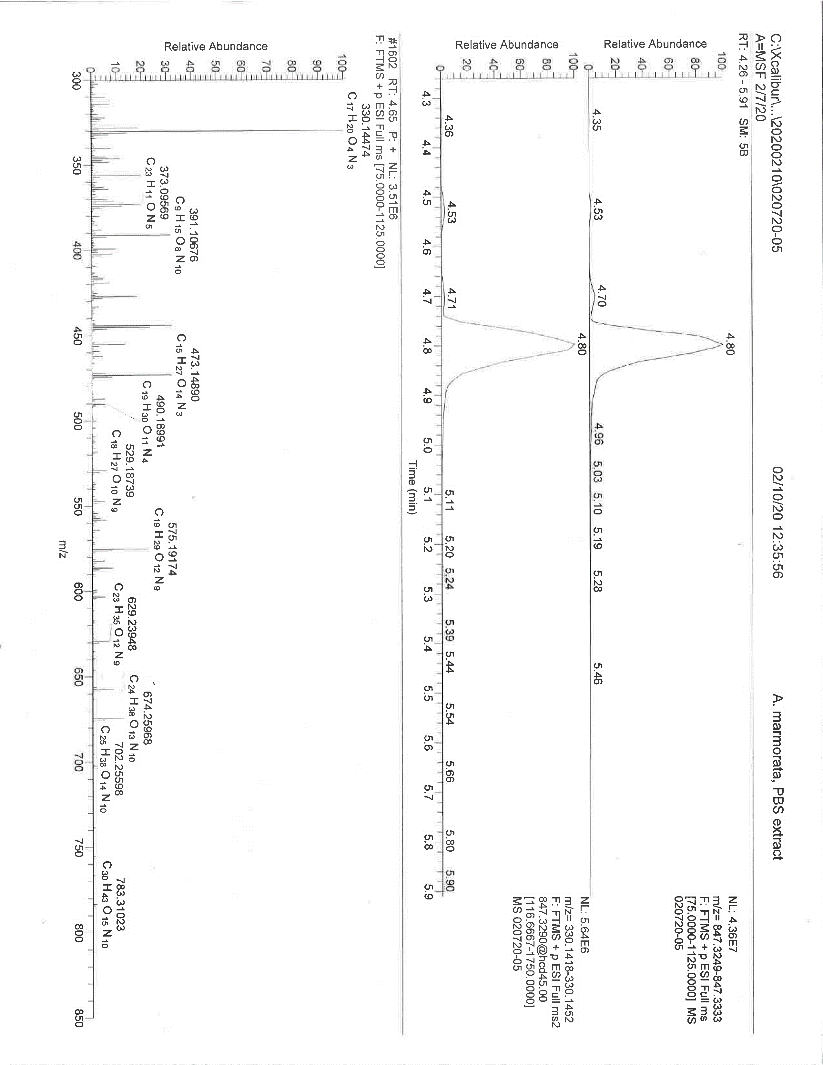 |
| phalloidin  (reference standard) | 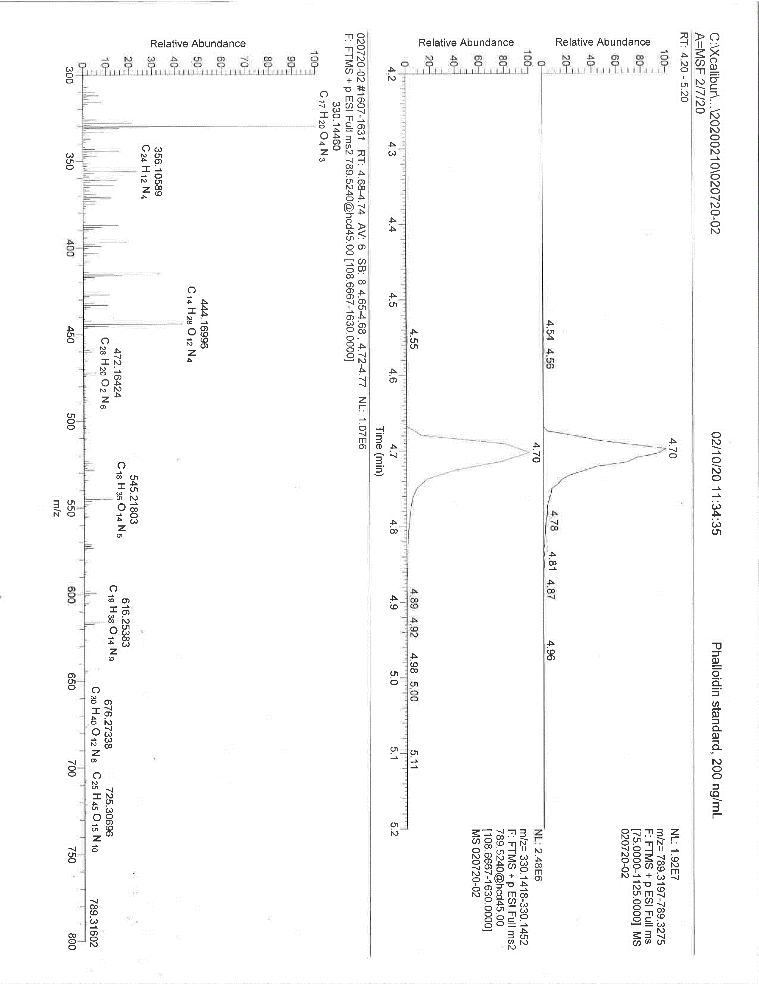 |
| phallacidin  (reference standard) | 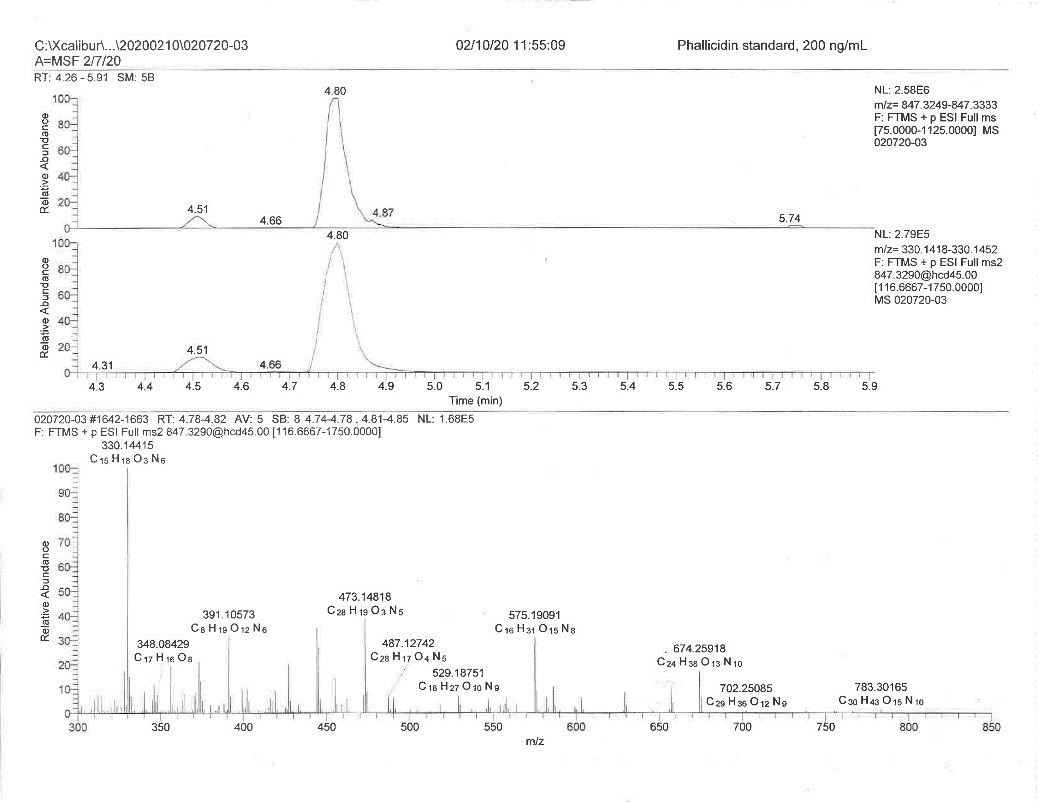 |
